# Supplementary material for: Linking emotional valence and anxiety in a mouse insula-amygdala circuit
Source: Nat Commun. 2023 Aug 21;14:5073. doi: 10.1038/s41467-023-40517-1 (PMC10442438; doi:10.1038/s41467-023-40517-1)
Supplement: Supplementary file 1 — Supplementary Information [file 41467_2023_40517_MOESM1_ESM.pdf]

Supplementary Online Material

Linking emotional valence and anxiety in a mouse insula-amygdala circuit.

Nicolas C, Ju A, Wu Y, Eldirdiri H, Delcasso S, Couderc Y, Fornari C, Mitra A, Supiot L, Vérité A, Masson M, Rodriguez-Rozada S, Jacky D, Wiegert JS, Beyeler A.

Experiments	***Coordinates AP/ML/DV from Bregma (mm)***	***Viral vector***	***Volume injected***	***Titer***	***Provider***
Glutamatergic specific fiber photometry	aIC: 1.7/3.1/-3.5<br>pIC:-0.35/4.0/-4.2	AAV9-CaMKIIα-GCaMP6f-WPRE	300 nL	$1.0x10^{13}$ vg/mL	Addgene
Projection specific fiber photometry	aIC: 1.7/3.1/-3.5	AAV9-syn-Flex-GCaMP6m-WPRE	300 nL	6.3x1013GC/mL	UPENN
	BLA:-1.6/3.3/-4.9	CAV2-Cre	300 nL	1.1x1013 pp/mL	IGMM
Projection mapping	BLA:-1.6/3.3/-4.9	AAV9-CaMKIIα-eYFP	300 nL	$2.2x10^{13}$ GC/mL	Addgene
Ex-vivo electro-physiology	IC: 0.0/4.0/-4.0	AAV9/2-CaMKIIα-hChR2(E123A)-eYFP-WPRE (ChETA 2.0 variant for ultrafast optogenetic control)	250 nL	8.8x1012GC/mL	UPENN
	BLA:-1.6/3.3/-4.9	CTB AF555	200 nL	NA	Fischer Scientific
	CeM:-0.8/2.35/-5.2	CTB AF647	100 nL	NA	Fischer Scientific
somBiPOLES optogenetic	aIC: 1.7/3.1/-3.5	AAV9-hSyn-DIO-somBiPOLES-mCerulean<br>or AAV$_9$-CAG-Flex-mCerulean	300 nL	$6.3x10^{14}$GC/mL	donated from Simon Wiegert
	BLA:-1.6/3.3/-4.9	CAV2-Cre<br>or AAVrg-hSyn-Cre	300 nL	$1.12x10^{13}$ pp/mL<br>$1.2x10^{13}$GC/mL	IGMM<br>Addgene
Optogenetic inhibition	aIC: 1.7/3.1/-3.5	AAV1-hSyn1-SIO-stGtACR2-FusionRed<br>or AAV1-hSyn-DIO-mCherry	300 nL	1.9x1013GC/mL<br>1.0x1013 vg/mL	Addgene<br>Addgene
	BLA:-1.6/3.3/-4.9	CAV2-Cre	300 nL	1.1x1013 pp/mL	IGMM
Optogenetic excitation	aIC: 1.7/3.1/-3.5	AAV5-Ef1a-DIO-hChR2(E123T/T159C)-eYFP<br>or AAV5-EF1a-DIO-eYFP	300 nL	$4x10^{12}$GC/mL	UNC GTC Vector core
	BLA:-1.6/3.3/-4.9	CAV2-Cre	300 nL	$1.12x10^{13}$ pp/mL	IGMM

<u>Supplementary Table 1</u>: Details of coordinates, neuronal tracing and viral vectors used for each experiment.

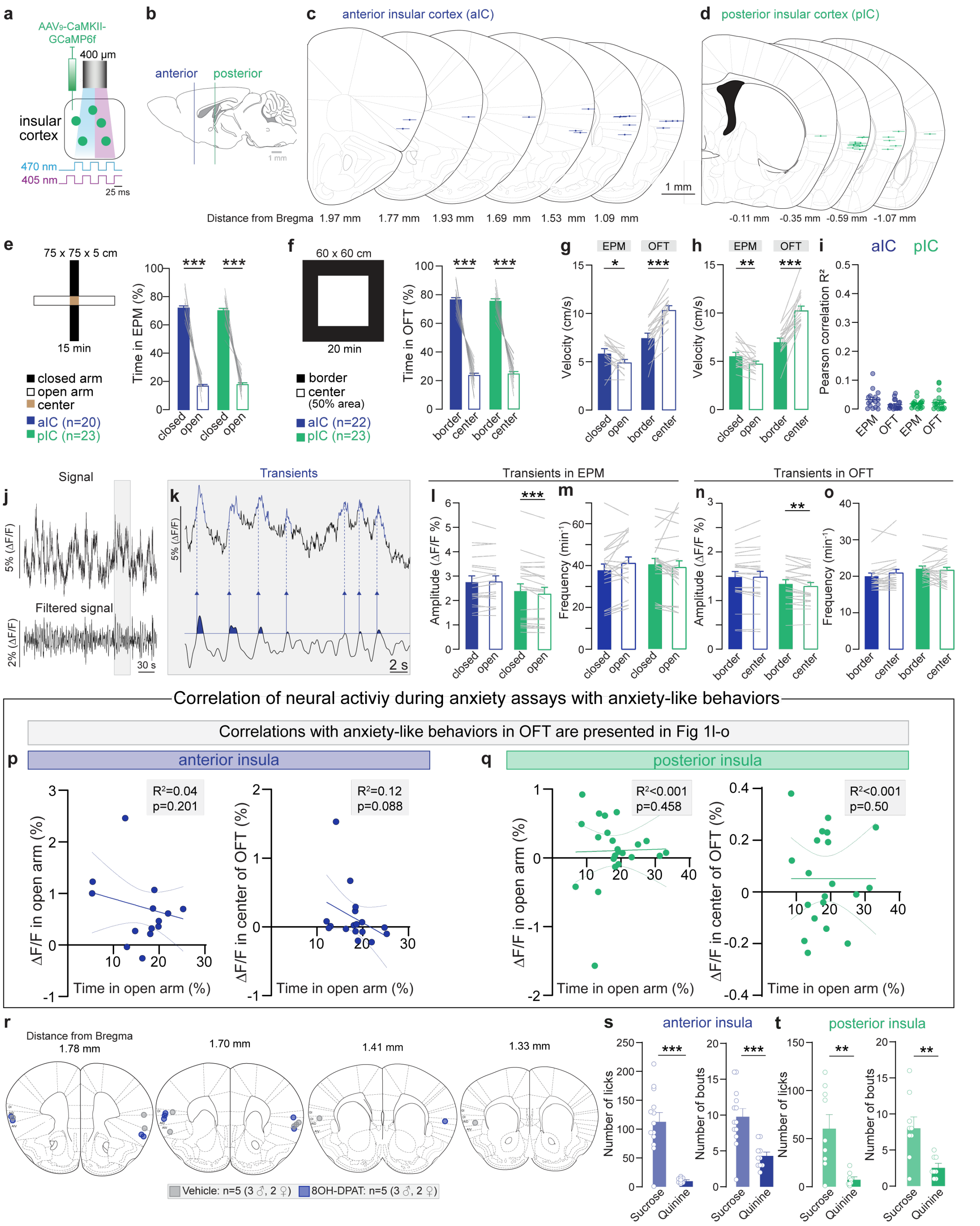

Supplementary Figure 1: **Histological verifications and complementary anxiety responses of aIC and pIC excitatory neurons**. **a**. Schematic of calcium imaging. **b.** Histology verification of fiber implantation sites in aIC (**c**) and pIC (**d**). **e**. Percentage of time spent in closed and open arms of the EPM (Two-way ANOVA, $F_{(1,41)}$=738.2, ***p<0.0001 for the arms (open, closed), no significant effect of the region (aIC, pIC) and no interaction, Bonferroni's post-hoc ***p<0.0001, aIC n=20 mice, pIC n=23 mice). **f.** Percentage of time spent

in borders and center of the OFT (Two-way ANOVA, $F_{(1,43)}$=465.4, ***p<0.0001 for the zone (border, center), no significant effect of the region (aIC, pIC) and no interaction, Bonferroni's post-hoc ***p<0.0001, aIC n=22 mice, pIC n=23 mice). **g-h.** Velocity in the open and close arms during EPM test and in the center and the border in the OFT for photometry recordings. **g.** Two-tailed paired t-test, t=2.599, *p=0.02, n=16 mice for EPM, two-tailed paired t-test, t=7.749, ***p<0.0001, n=18 mice for OFT. **h.** Two-tailed paired t-test, t=3.356, **p=0.0037, n=18 mice for EPM, two-tailed paired t- test, t=8.957, ***p<0.0001, n=18 mice for OFT. **i.** Pearson correlation of the velocity in the EPM (n=16 mice for aIC, n=18 mice for pIC) and the OFT (n=18 mice for aIC and pIC) with the respective calcium signal of aIC and pIC glutamatergic neurons. **j.** Fiber photometry signal recorded from aIC neurons, (Top) Bulk GCaMP6f signal, and (Bottom) filtered GCaMP6f signal for calcium transient detection. ΔF/F represents the fluorescent changes from the mean level of the entire recording time series. **k.** Representation of automated transient detection. Filtered GCaMP6f peaks exceeding the threshold (horizontal line in the lower trace) were identified as the transients. **l.** Average calcium transient amplitude of aIC (n=20 mice) and pIC (n=23 mice) between open and closed arms. (Two-way ANOVA, $F_{(1,41)}$=5.618, *p= 0.023 for the arms (open, closed), $F_{(1,41)}$=8.176, **p= 0.007 for the arms x region (aIC, pIC) interaction without effect of the region. Bonferroni's post-hoc show a lower transient amplitude in the open compared to the close arms for pIC, ***p=0.0009). **m.** Average calcium transient frequency of aIC (n=20 mice) and pIC (n=23 mice) between open and closed arms. (Two-way ANOVA, $F_{(1,41)}$=5.476, *p= 0.024 for the arms (open, close) x region (aIC, pIC) interaction without effect of the arms and the region). **n**. Average calcium transient amplitude of aIC (n=22 mice) and pIC (n=23 mice) between center and border of the OFT. (Two-way ANOVA, $F_{(1,43)}$=4.906, *p= 0.032 for the zone (border, center) and a trend for zone x region (aIC, pIC) interaction, $F_{(1,43)}$=3.866, p= 0.056, with no effect of the region. Bonferroni's post-hoc show a lower transient amplitude in the center than the border for the pIC, **p=0.009). **o**. Average calcium transient frequency of aIC (n=22 mice) and pIC (n=23 mice) between center and border of the OFT. (Two-way ANOVA, trend for the interaction zone (center, border) x region (aIC, pIC), $F_{(1,43)}$=3.750, p= 0.059; with no effect of the zone or the region). **p-q**. Correlation of aIC and pIC calcium signal in anxiogenic spaces of the EPM and OFT (e.g. EPM open arms and OFT center), with the time mice spent in the center of the EPM (Two-tailed Pearson correlation). **r**. Histology verification of internal cannula in aIC. **s**. Average number of sucrose and quinine licks (Left, two-tailed unpaired t-test, t=5.906, ***p<0.001, n=13 mice for sucrose, n=10 mice for quinine) and average number of sucrose and quinine bouts (Right, two-tailed unpaired t-test, t=3.943, ***p<0.001, n=13 mice for sucrose, n=10 mice for quinine) performed during recording of aIC glutamatergic neurons. **t**. Average number of sucrose and quinine licks (Left, two-tailed unpaired t-test, t=3.535, **p=0.003, n=9 mice for sucrose, n=8 mice for quinine) and average number of sucrose and quinine bouts (Right, two-tailed unpaired t-test, t=3.327, **p=0.005, n=9 mice for sucrose, n=8 mice for quinine) performed during recording of pIC glutamatergic neurons. All the results are represented as mean ± SEM.

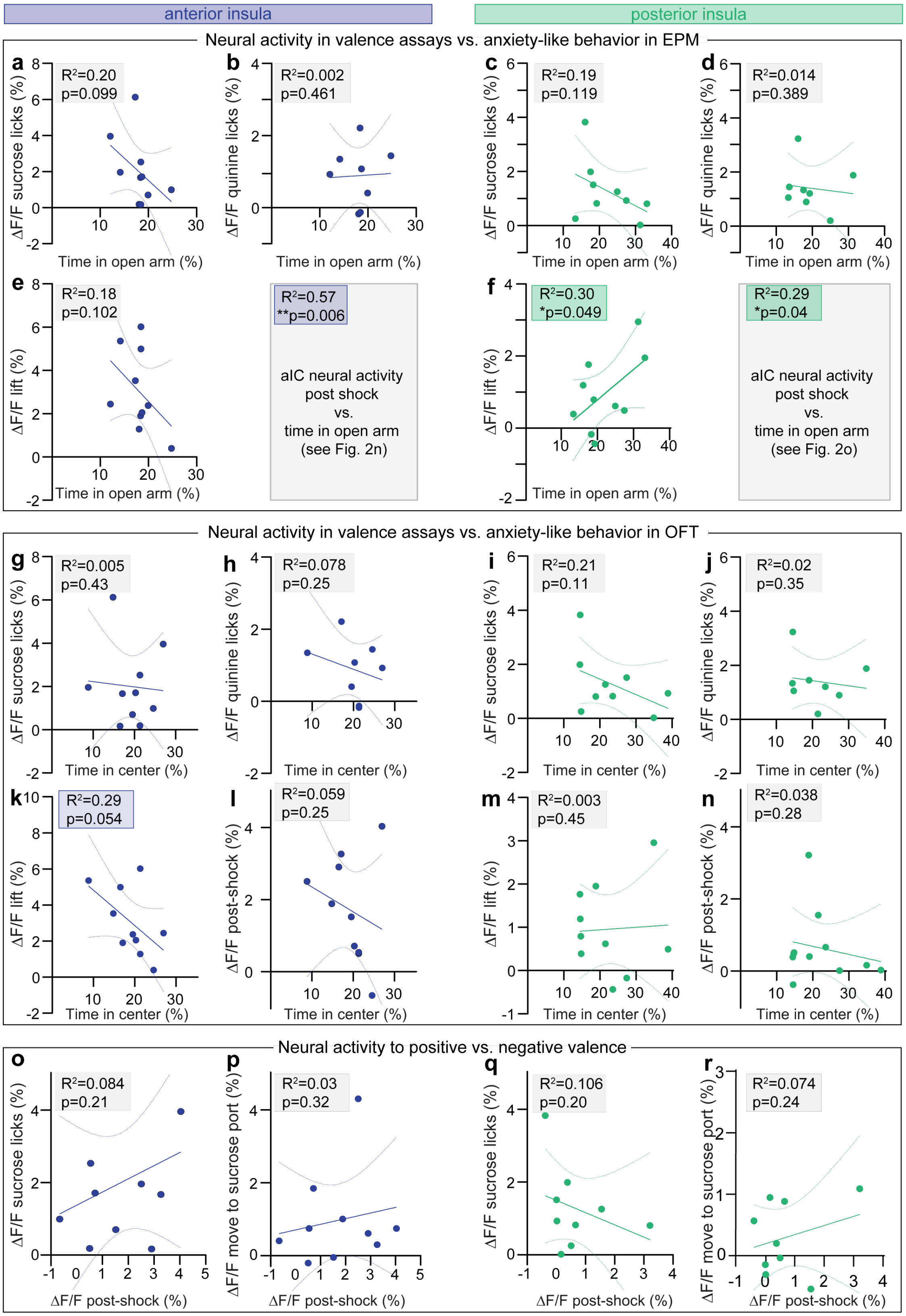

Supplementary Figure 2: **Correlation of the activity of excitatory neurons in the anterior and posterior insular cortex (aIC and pIC) with anxiety-related behaviors (a-n) or with neural activity in other tests (o-r).** Pearson correlations were performed using GraphPad Prism 9.

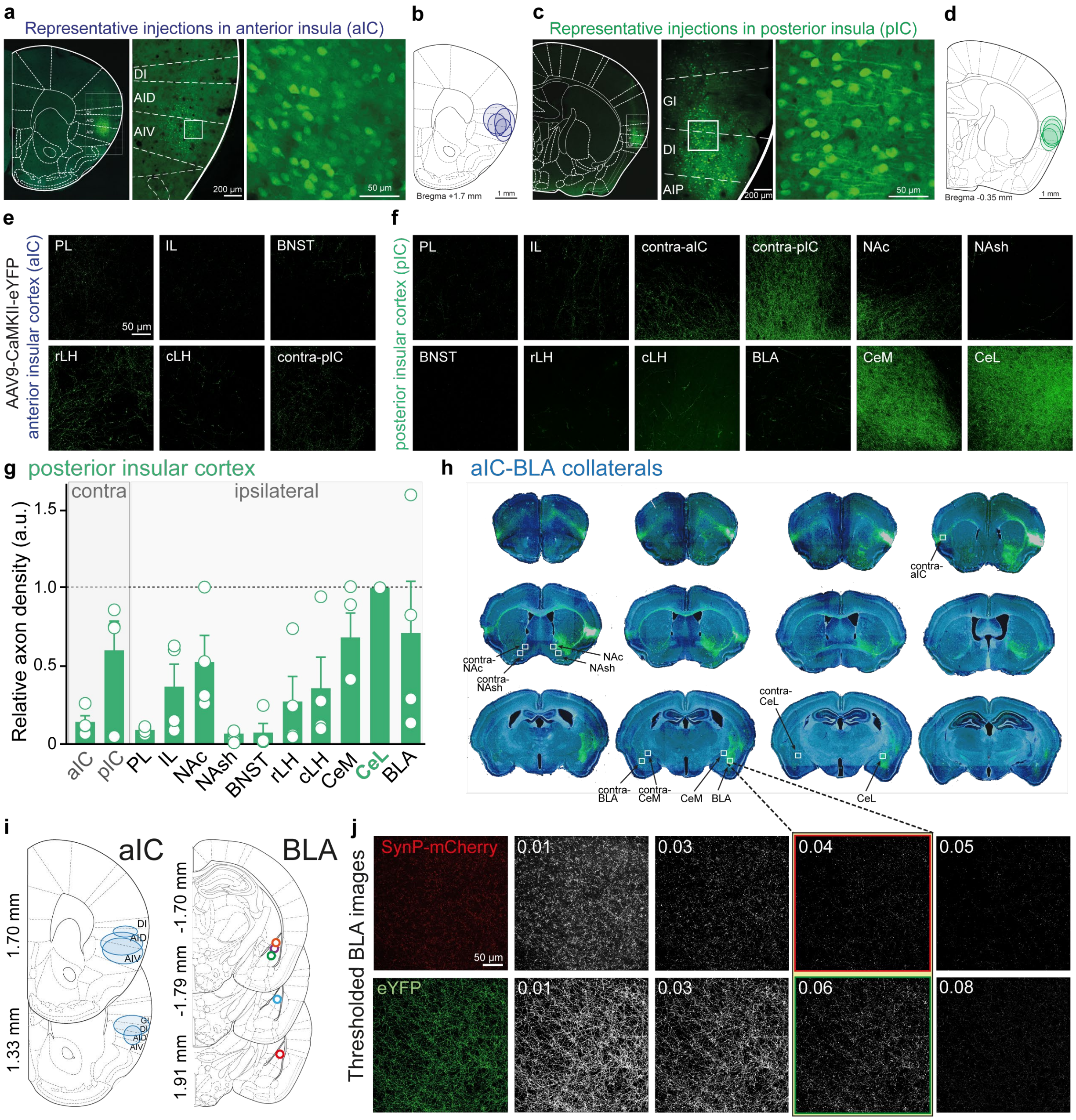

Supplementary Figure 3: Histology of injections in the insular cortex for the anatomical mapping. a, c. Representative images of glutamatergic neurons expressing eYFP in aIC and pIC. **b, d.** Schematic representing the spread of AAV9-CaMKIIα-eYFP injections in aIC and pIC of the 8 animals included in the anatomical study. **e, f.** Representative image of eYFP+ axonal projections from one mouse expressing CaMKIIα-eYFP in neurons of aIC (left) or pIC (right). **g.** Number of fluorescent pixels normalized to the average value of the maximal projection region (CeL) per image from pIC neurons (n= 4 mice). **h.** Example of a slide showing different coronal brain slices (top left: rostral, bottom right: caudal) to illustrate the imaging and mapping of aIC-BLA collaterals. Blue represents DAPI staining. Green represents AAV9-DIO-eYFP viral infection. **i.** Histological verification of the cre-dependent dual-vector AAV9-DIO-eYFP + AAV8/2-DIO-SynP-mCherry injection in the aIC (left) and the CAV2-cre injection in the BLA (right). Circles on the left represent the spread of the viral vector in the aIC. Antero-posterior levels (distance from Bregma, in millimeters) are indicated at the left of each slice. **j.** Examples of fluorescence detection with different

thresholds for SynP-mCherry and eYFP detection. We chose 0.04 for SynP-mCherry and 0.06 for eYFP. All the results are represented as mean ± SEM.

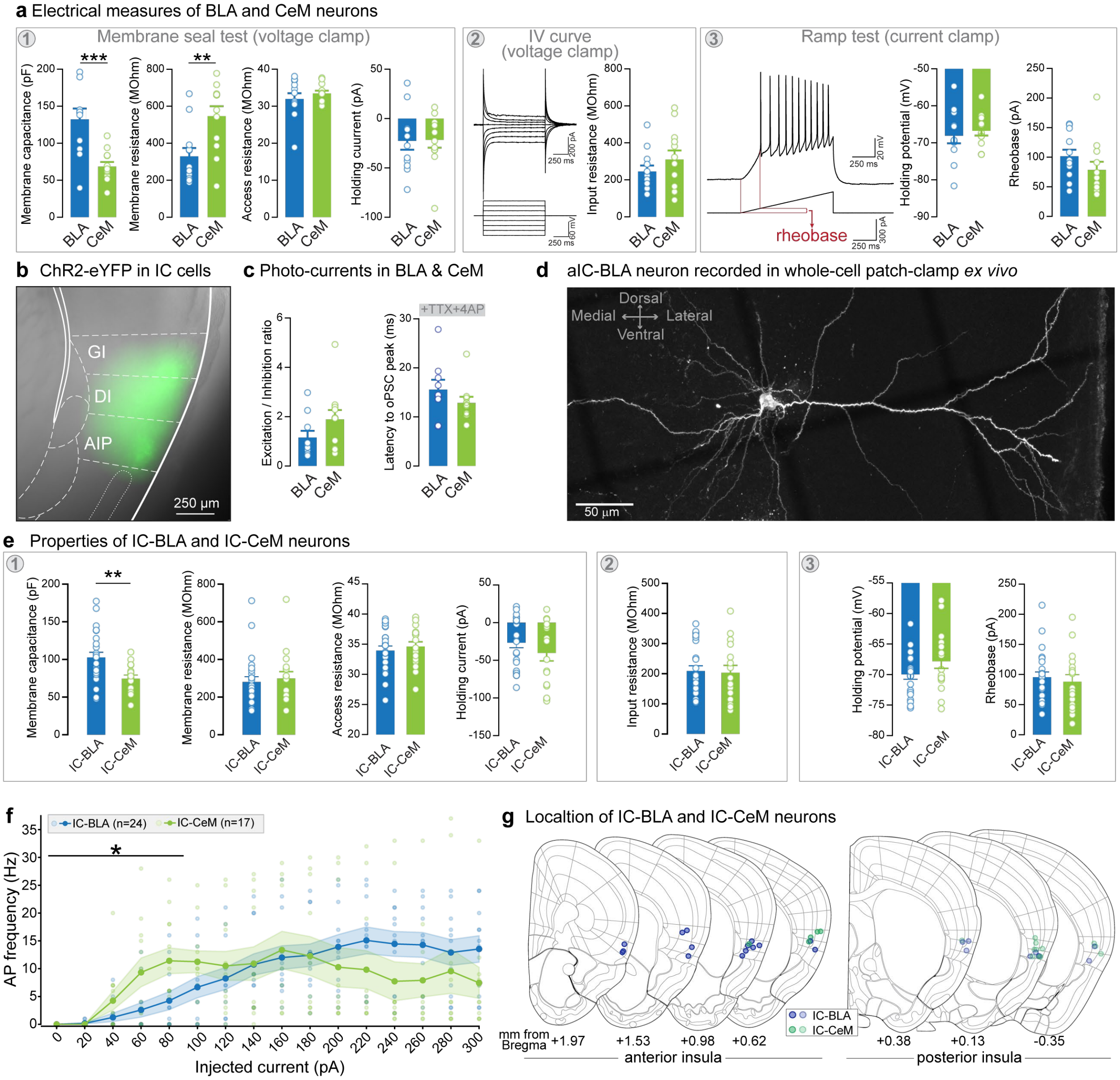

Supplementary Figure 4: **Electrophysiological properties of recorded neurons**. **a**. Intrinsic properties of neurons in the BLA and CeM recorded for synaptic properties upon the optogenetic activation of the insular terminals in Fig. 4a-g (n=12 cells for BLA and CeM). Measures were obtained from the membrane seal test [1], the IV curve [2], and the Ramp test [3]. Membrane capacitance and resistance were higher for BLA compared to CeM neurons (Two-tailed unpaired t-test, t=4.058, ***p=0.0005, and t=3.129, **p=0.0049 respectively). **b**. Representative images of CTB injection sites in BLA and CeM. **c**. Excitation/inhibition ratio and the latency to oEPSC peak by TTX and 4AP application from IC photo-current in the BLA and CeM (BLA n= 9-10 cells, CeM n=10-11 cells). **d**. Confocal image of biocytin-filled neurons in the insula. **e.** Membrane capacitance was higher for IC-BLA neurons compared to IC-CeM neurons (Two-tailed unpaired t-test, t=3.083, **p=0.0038, IC-BLA n=24 cells, IC-CeM n=17 cells). **f.** Action potential frequency was higher for small injected currents (0-80 pA) in IC-CeM compared to IC-BLA neurons (repeated measures ANOVA $F_{(4,156)}$=2.661, *p=0.037). **g**. Location of patched cells from IC-BLA and IC-CeM neurons

on Allen brain atlas. The anterior-posterior (AP) distances from the bregma (mm), corresponding to Paxinos brain atlas, are written under the atlas. For determining the AP coordinate to divide aIC and pIC, we calculated the middle of AP level from starting and end of the insular cortex according to Paxinos atlas, which was AP +0.62 mm from the bregma. The number of IC-BLA neurons was 24 (aIC-BLA n=14-15 cells and pIC-BLA n=9 cells; in layer 2/3 n=11-12 cells and in layer 5 n=12 cells) and of IC-CeM neurons was 17 (aIC-CeM n=5 cells and pIC-CeM n=11-12 cells; in layer 2/3 n=5-6 cells and in layer 5 n=10-11 cells). All the results are represented as mean ± SEM.

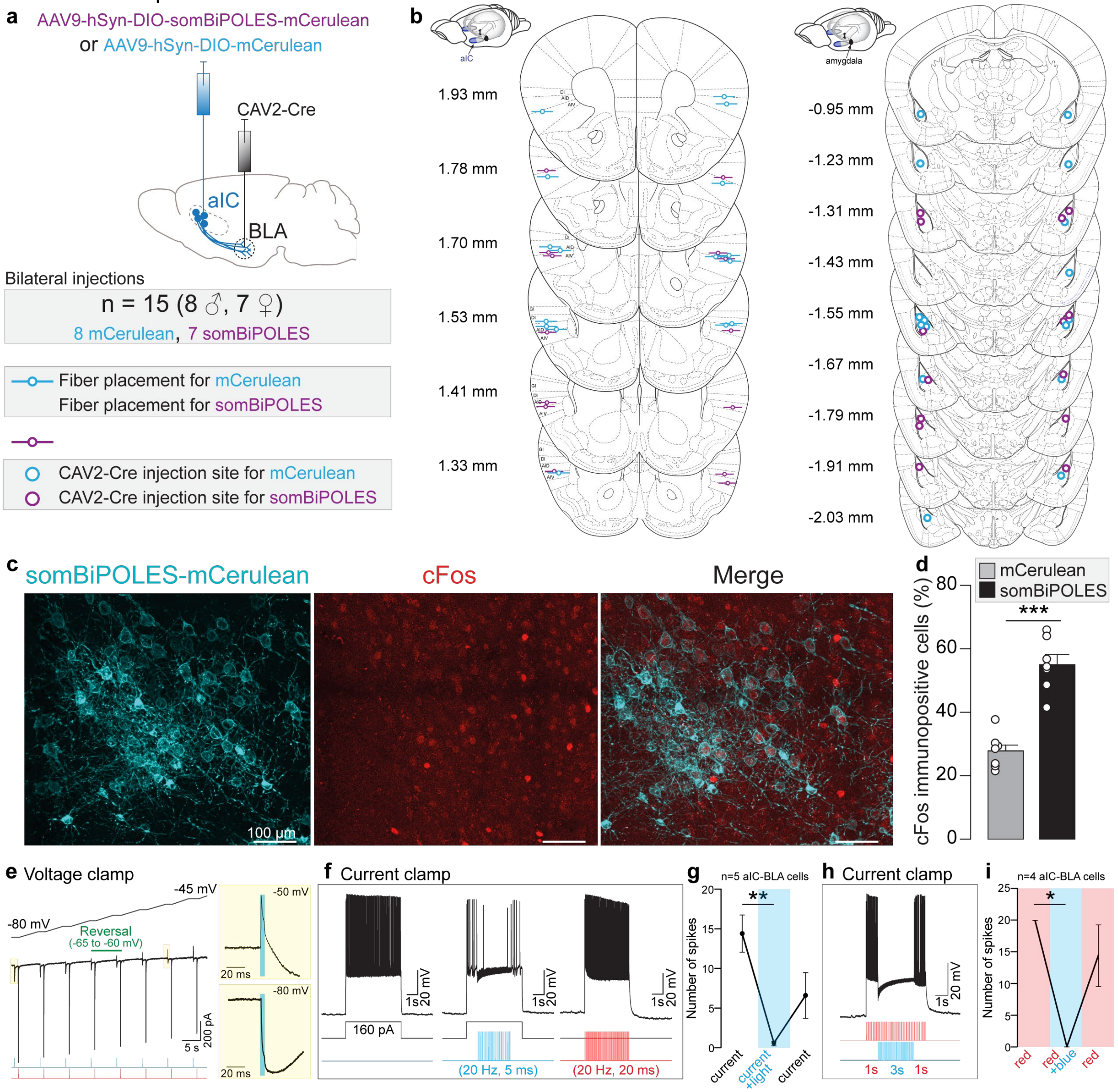

Supplementary Figure 5: **Histological verification of viral vector injections and optic fiber placement for somBiPOLES optogenetic experiments. a.** Experimental design and legend for panel b. **b.** Location of the fiber implants and retrograde viral vector injection. Bars represent the optic fiber tip in the aIC. Circles represent CAV2-Cre injection site in the BLA. Antero-posterior levels (distance from Bregma, in millimeters) are indicated at the left of each slice. **c.** Fluorescent images of neurons expressing somBiPOLES-mCerulean, cFos and the merge of the two images. **d**. Percentage of cFos-immunoreactive cells is higher in aIC neurons projecting to BLA expressing somBiPOLES compared to the control mCerulean (two-tailed unpaired t-test, t=7.612, ***p<0.0001, mCerulean n=8 mice, somBiPOLES n=7 mice). **e**. Representative whole-cell patch-clamp recordings in voltage-clamp mode. **f.** Qualitative representation of aIC-BLA neuron in current-clamp mode spiking to a 160 pA step current (left trace), blue light-activated G*t*ACR2 leads to efficient inhibition of a step current evoked spiking (middle trace), and Chrimson channel activation leads to robust spiking without current stimulation (right trace). **g**. The number of spikes of aIC-BLA neurons

during blue light stimulation inducing G*t*ACR2 activation decreased compared to non-light stimulation (Non-parametric one-way ANOVA, p=0.0008, Dunn's post hoc **p=0.0031). **h**. Representative trace in current-clamp mode showing G*t*ACR2-assisted inhibition to an ongoing Chrimson-induced action potential. **i**. The number of spikes of aIC-BLA neurons during simultaneous stimulation of G*t*ACR2 and Chrimson channels by red and blue lights (non-parametric one way ANOVA, p=0.037, Dunn's post-hoc *p=0.0431). AID: agranular insula, AIV: agranular insula, DI: dysgranular insula, GI: granular insula, BLA: basolateral amygdala. Coronal sections from Franklin and Paxinos[6]. All the results are represented as mean ± SEM.

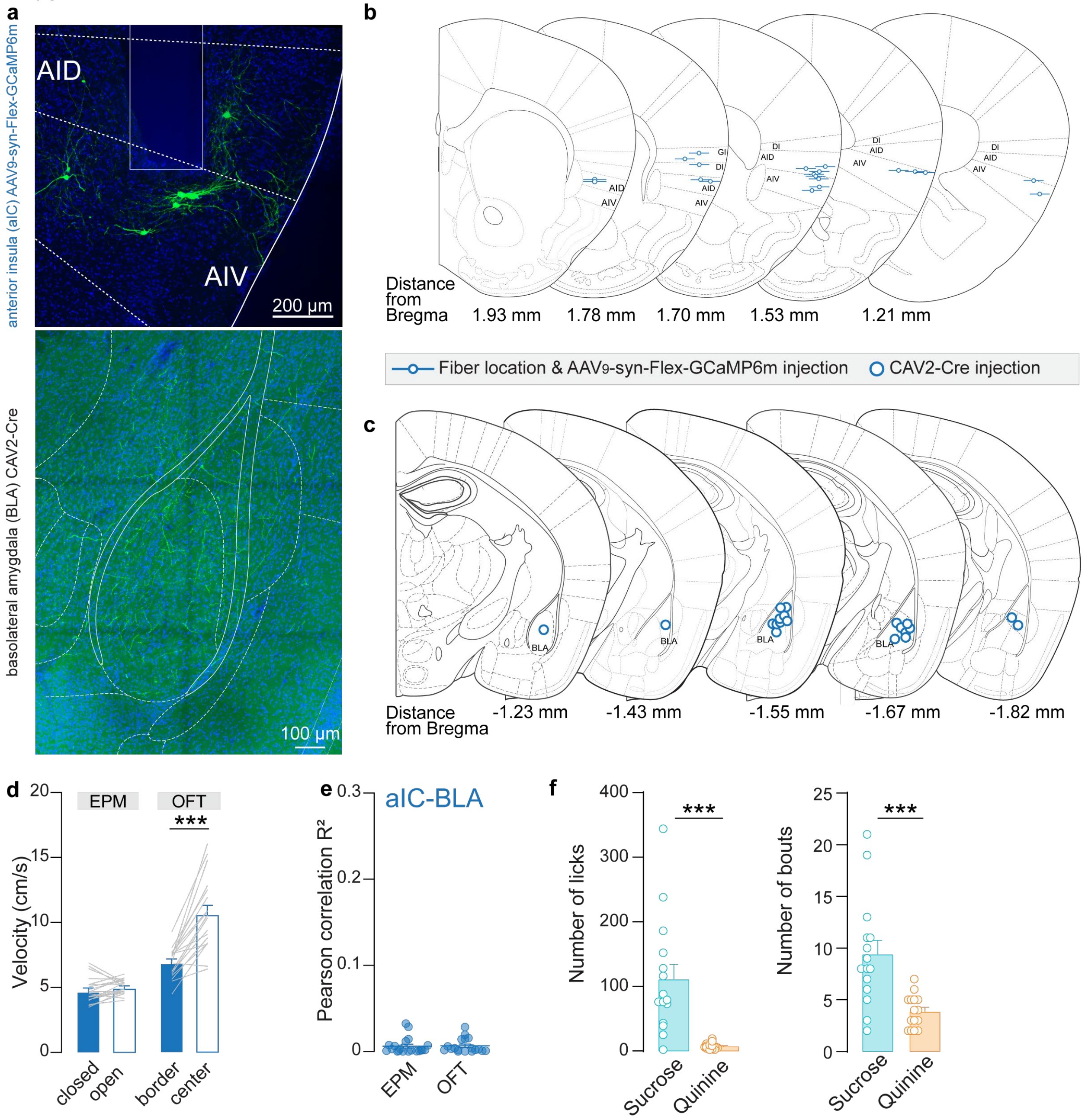

<u>Supplementary Figure 6</u>: Histological verification of viral injections and optic fiber placement from fiber photometry experiments. a. Representative image of aIC to BLA neurons expressing GCaMP6m into the aIC with the optic fiber track (top). Fibers expressing GCaMP6m into the BLA (bottom). **b.** Bars represent the optic fiber tip in the aIC for mice who received injection of the virus carrying the gene coding for GCaMP6m. **c.** Circles represent CAV2-Cre injection site in the BLA. Antero-posterior levels (distance from Bregma, in millimeters) are indicated at the bottom of each slice. **d**. Velocity in the closed and open arms of EPM. In OFT, the velocity was higher in the center compared to the border (two tailed paired t-test,

t=6.879, ***p<0.0001, n=18 mice). **e**. There is no correlation of aIC-BLA activity with locomotor instantaneous speed within the EPM (n=20 mice) and OFT (n=18 mice). **f**. Number of sucrose and quinine licks (Left, two-tailed unpaired t-test, t=4.471, ***p=0.0001, n=15 mice for both sucrose and quinine) and average number of sucrose and quinine bouts (Right, two-tailed unpaired t-test, t=3.916, ***p=0.0005, n=15 mice for both sucrose and quinine) performed during aIC-BLA calcium signal recording. Coronal sections from Franklin and Paxinos[6]. AID: agranular insula, AIV: agranular insula, DI: dysgranular insula, GI: granular insula, BLA: basolateral amygdala. All the results are represented as mean ± SEM.

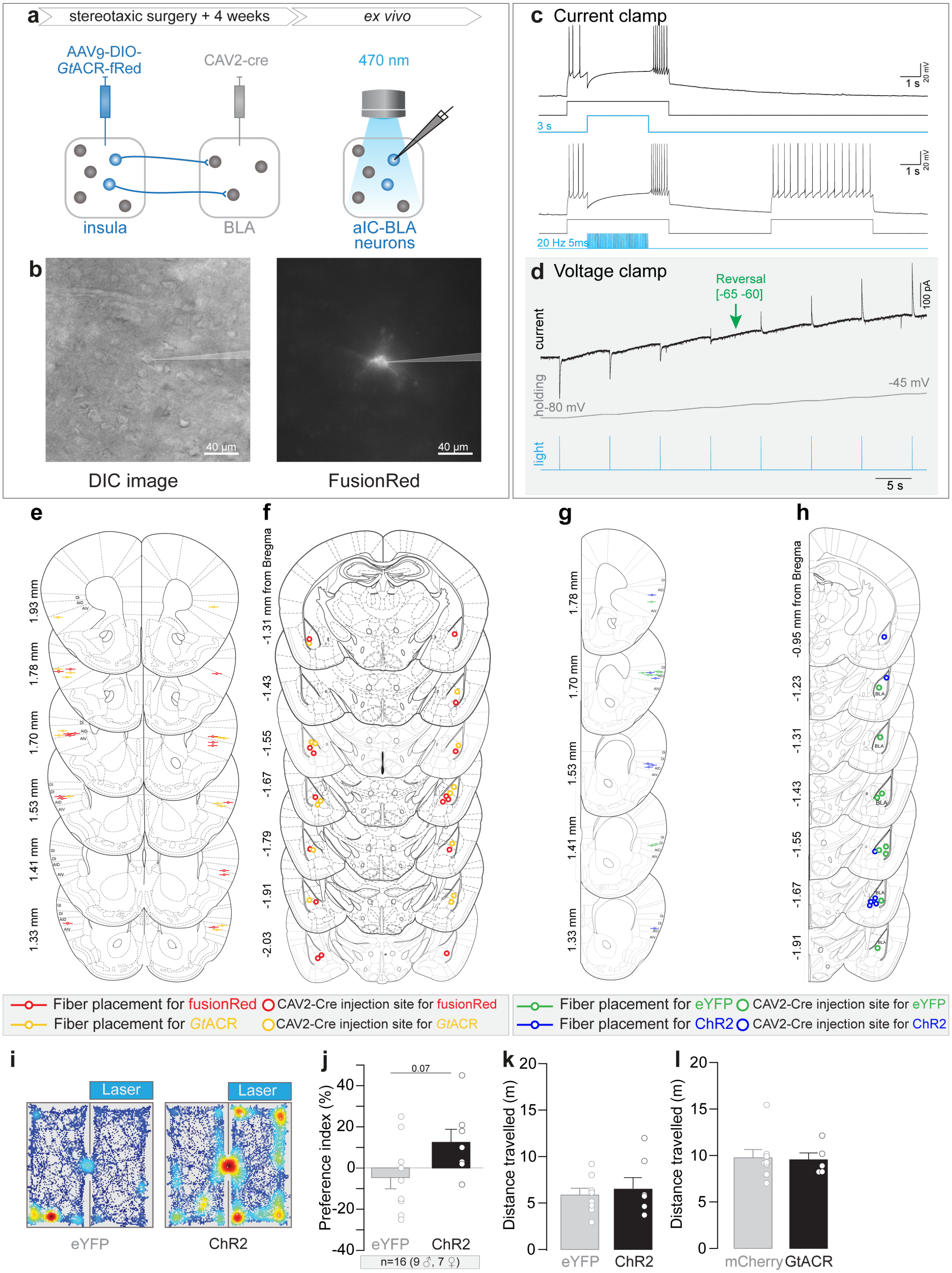

a
stereotaxic surgery + 4 weeks
ex vivo
AAV9-DIO-GtACR-fRed
CAV2-cre
470 nm
insula
BLA
aIC-BLA neurons
b
40 µm
40 µm
DIC image
FusionRed
c Current clamp
1 s
20 mV
3 s
20 Hz 5ms
d Voltage clamp
Reversal [-65 -60]
current
holding
-80 mV
-45 mV
100 pA
light
5 s
e
f
g
h
1.93 mm
1.78 mm
1.70 mm
1.53 mm
1.41 mm
1.33 mm
-1.31 mm from Bregma
-1.43
-1.55
-1.67
-1.79
-1.91
-2.03
-0.95 mm from Bregma
-1.23
Fiber placement for fusionRed
CAV2-Cre injection site for fusionRed
Fiber placement for GtACR
CAV2-Cre injection site for GtACR
Fiber placement for eYFP
CAV2-Cre injection site for eYFP
Fiber placement for ChR2
CAV2-Cre injection site for ChR2
i
Laser
Laser
eYFP
ChR2
j
0.07
Preference index (%)
eYFP
ChR2
n=16 (9 ♂, 7 ♀)
k
Distance travelled (m)
eYFP
ChR2
l
Distance travelled (m)
mCherry
GtACR

<u>Supplementary Figure 7</u>: Histological verification of viral vector injections and optic fiber placement for optogenetic experiments. a. Injection of a viral vector carrying *Gt*ACR2 gene in the aIC. After 6 weeks, the brain was sectioned into acute slices for whole-cell patch-clamp recordings. **b**. Left: differential interference contrast (DIC) image of a 300μm thick coronal brain section of a mouse expressing *Gt*ACR2-fRed in aIC-BLA projectors. Right: aIC-BLA projectors expressing *Gt*ACR2-fRed and recorded in whole-cell parch clamp. **c**. Representative whole-cell patch-clamp recordings in current-clamp mode of a *Gt*ACR2-expressing cell silenced by light application. Illuminating with 473 nm light delivered for 3 seconds or in 5 ms pulses at 20 Hz inhibits action potential generation induced by a current injection. The cell firing returned to normal current-induced firing pattern after light stimulation. **d**. Representative whole-cell patch-clamp recordings in voltage-clamp mode. **e**. Bars represent the optic fiber tip in the aIC of mice who received injection of the virus carrying the gene coding for *Gt*ACR2 and the control virus fusionRed. **f**. Circles represent CAV2-Cre injection site in the BLA for *Gt*ACR2 experiment. **g.** Bars represent the optic fiber tip in the aIC for mice who received injection of the virus carrying the gene coding for ChR2 and the control virus eYFP. **h.** Circles represent CAV2-Cre injection site in the BLA for ChR2 experiment. Antero-posterior levels (distance from Bregma, in millimeters) are indicated at the left side of each slice. AID: agranular insula, AIV: agranular insula, DI: dysgranular insula, GI: granular insula, BLA: basolateral amygdala. Coronal sections from Franklin and Paxinos[6] **i**. Representative traces showing an occupancy heatmap of the time spent in the non-stimulated (left) or stimulated (right) side for a control eYFP mouse (left) and ChR2 mouse (right). **j**. Preference index calculated as the time difference between the stimulation side and non-stimulation side, divided by total time spent and multiplied by 100. ChR2 group tends to spend more time on the stimulation side in comparison to the control group (two-tailed unpaired t-test, t=1.929, p=0.074, eYFP n= 7 mice, ChR2 n=7 mice). **k**. Average distance travelled during the RTPP test for eYFP (n=8 mice) and ChR2 (n=7 mice) groups (Two-tailed unpaired t-test, t=0.553, p=0.59). **l**. Average distance travelled during the RTPP test for mCherry (n=8 mice) and GtACR (n=8 mice) groups (two-tailed unpaired t-test, t=0.167, p=0.87). All the results are represented as mean ± SEM.

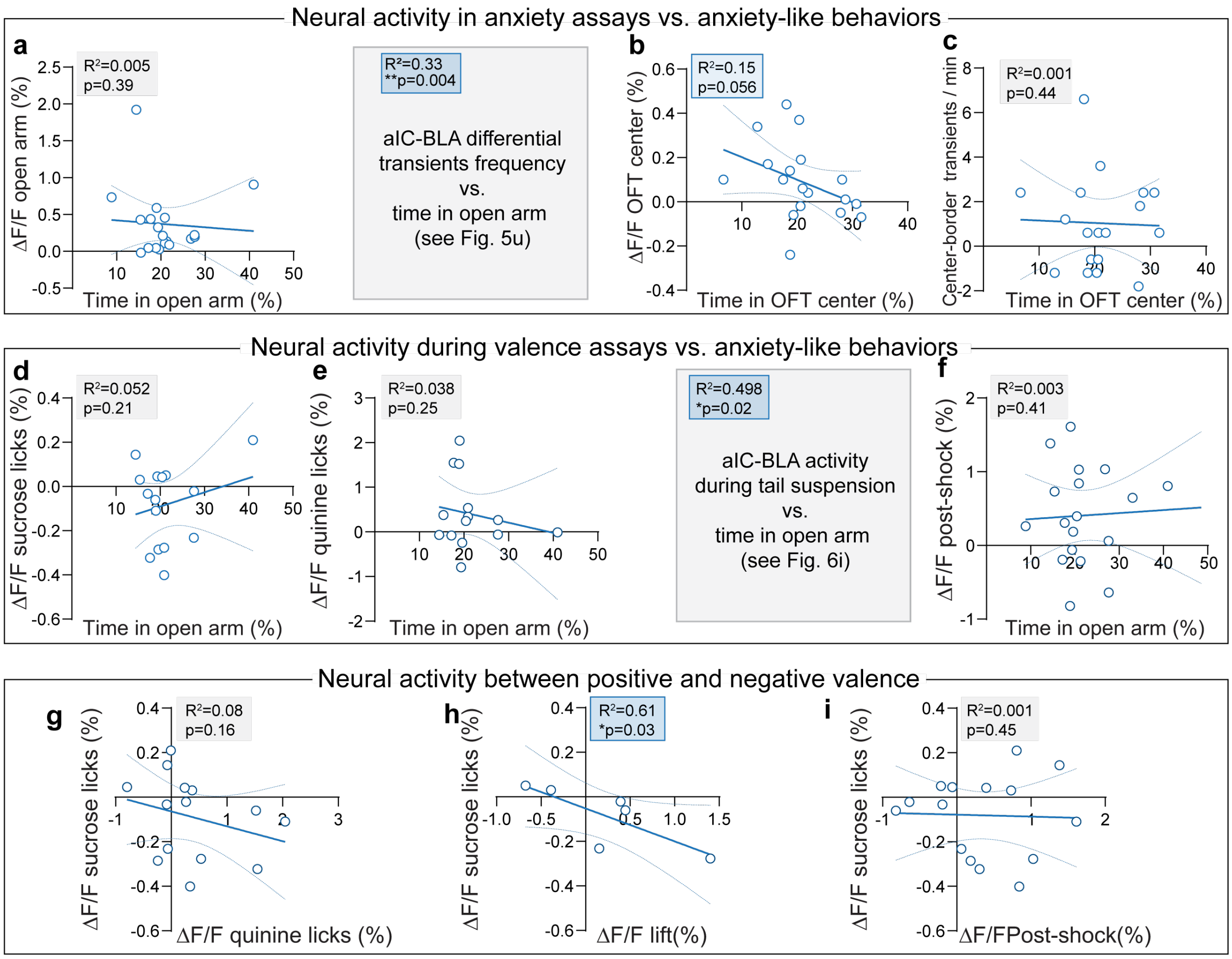

j

		calcium photometryimaging aIC	pIC	aIC-BLA	inhibition aIC	activation aIC-BLA	inhibition aIC-BLA
EPM open arm		↑	↔	↑	↑	↓	↓
OFT center		↑	↔	↑	tbd	↓	↓
Valence +	sucrose	↑	↑	↓	tbd	RTPP	RTPP
Valence -	quinine	↔	↑	↔	tbd	tbd	tbd
	suspension	↑	↔	↑			
	footshock	↑	↔	↑			

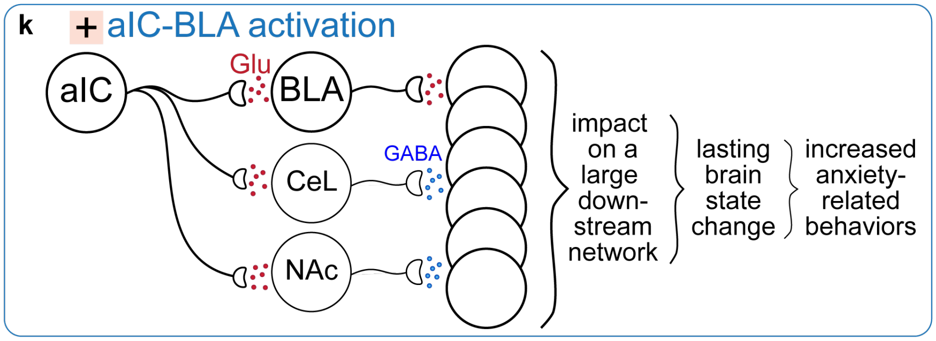

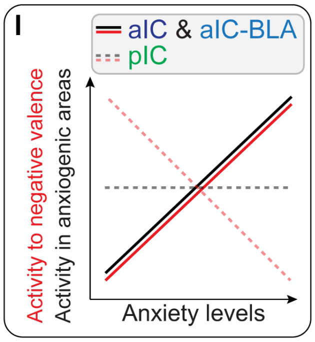

Supplementary Figure 8: Correlation of aIC-BLA neural activity with anxiety-like behaviors and across behaviors. a. Calcium signal recorded when mice are in the open arms of the EPM is not correlated with the total time mice spend in the location. **b.** The signal recorded when mice are in the center of the OFT tends to be negatively correlated with the total time mice spent in this area. **c.** The difference of transient frequency between the center and border of the OFT is not correlated with the total time mice spend in the center of the OFT. **d-f.** Calcium signal during the tail suspension is negatively correlated with the time mice spent in the open arm of the EPM, but not with the response to sucrose, quinine or footshock. **g-i.** Calcium signal during sucrose consumption is negatively correlated with the calcium signal recorded during tail suspension, but not during quinine licking or foot-shock. **j.** Summary diagram of all photometry, pharmacology and optogenetics observation presented in the manuscript. **k.** Circuit model of persistent behavioral effects induced by aIC-BLA optogenetic activation (Figure 5a-f). **l.** Summary diagram representing the positive correlation observed between the aIC-BLA neural response to anxiogenic area and negative valence, with the levels of anxiety, based on Fig. 1l-o, Supplementary Data Fig 2e-f, k, Fig. 5u, Supplementary Data Fig. 8b and Fig. 6i. All correlations were analyzed by one-tailed Pearson correlation.
