# Supplementary material for: Linking emotional valence and anxiety in a mouse insula-amygdala circuit
Source: Nat Commun. 2023 Aug 21;14:5073. doi: 10.1038/s41467-023-40517-1 (PMC10442438; doi:10.1038/s41467-023-40517-1)
Supplement: Supplementary file 3 — Description of Additional Supplementary Files [file 41467_2023_40517_MOESM3_ESM.pdf]

Description of Additional Supplementary Files

File Name: Supplementary Movie 1
Description: **aIC glutamatergic neuron activity in the elevated plus maze test (EPM).** Real-time recording of calcium signal in aIC neurons of a representative mouse injected with AAV_9-CaMKIIα-GCaMP6f-WPRE in the aIC, during exploration of the EPM.

File Name: Supplementary Movie 2
Description: **aIC-BLA neuronal activity in the elevated plus maze test (EPM).** Real-time recording of calcium signal in aIC-BLA neurons of a representative mouse injected with AAV_9-syn-Flex-GCaMP6m-WPRE in the aIC and CAV2-Cre in the BLA, during exploration of the EPM.
