# Supplementary material for: Linking emotional valence and anxiety in a mouse insula-amygdala circuit
Source: Nat Commun. 2023 Aug 21;14:5073. doi: 10.1038/s41467-023-40517-1 (PMC10442438; doi:10.1038/s41467-023-40517-1)
Supplement: Supplementary file 6 — Reporting Summary [file 41467_2023_40517_MOESM6_ESM.pdf]

nature portfolio

Corresponding author(s): Anna Beyeler

Last updated by author(s): Jul 10, 2023

Reporting Summary

Nature Portfolio wishes to improve the reproducibility of the work that we publish. This form provides structure for consistency and transparency in reporting. For further information on Nature Portfolio policies, see our Editorial Policies and the Editorial Policy Checklist.

Statistics

For all statistical analyses, confirm that the following items are present in the figure legend, table legend, main text, or Methods section.

n/a	Confirmed	
☐	☒	The exact sample size (*n*) for each experimental group/condition, given as a discrete number and unit of measurement
☐	☒	A statement on whether measurements were taken from distinct samples or whether the same sample was measured repeatedly
☐	☒	The statistical test(s) used AND whether they are one- or two-sided<br>*Only common tests should be described solely by name; describe more complex techniques in the Methods section.*
☐	☒	A description of all covariates tested
☐	☒	A description of any assumptions or corrections, such as tests of normality and adjustment for multiple comparisons
☐	☒	A full description of the statistical parameters including central tendency (e.g. means) or other basic estimates (e.g. regression coefficient) AND variation (e.g. standard deviation) or associated estimates of uncertainty (e.g. confidence intervals)
☐	☒	For null hypothesis testing, the test statistic (e.g. *F*, *t*, *r*) with confidence intervals, effect sizes, degrees of freedom and *P* value noted<br>*Give P values as exact values whenever suitable.*
☒	☐	For Bayesian analysis, information on the choice of priors and Markov chain Monte Carlo settings
☐	☒	For hierarchical and complex designs, identification of the appropriate level for tests and full reporting of outcomes
☐	☒	Estimates of effect sizes (e.g. Cohen's *d*, Pearson's *r*), indicating how they were calculated

Our web collection on statistics for biologists contains articles on many of the points above.

Software and code

Policy information about availability of computer code

Data collection	MATLAB (R2018, Mathworks) custom-written codes for fiber photometry and optogenetic manipulation during behavior experiments<br>Arduino custom-written code for optogenetic manipulation during behavior experiments<br>LAS Z 3.5.7 software for confocal microscopy (Leica SP8 microscope) for collection of the anatomical mapping data<br>LAS X software (Version 3.4.2) for fluorescence microscopy with THUNDER Imager 3D Tissue (Leica Microscopy) for histological verification<br>Digidata 1440 and pClamp11 software (Molecular Devices) for acquisition of electrophysiological recordings
Data analysis	Bonsai (2.6.3) for detecting mouse location during behavior experiments<br>MATLAB (R2018, Mathworks) for fiber photometry analysis, optogenetic experiments and anatomical mapping available at https://github.com/beyelerlab/FiberPhotometry_anxiety.git<br>Fiji (Image J, Java 1.8.0_172, NIH) for cFos counting<br>Adobe illustrator (Version 25.0.1) for verification of the viral expression and fiber implant location by overlaying atlas sections (Paxinos)<br>Clampfit software (Molecular Devices) for analysis of electrophysiological recording<br>Prism 9 (GraphPad, Version 9.0.0) for statistical analysis

For manuscripts utilizing custom algorithms or software that are central to the research but not yet described in published literature, software must be made available to editors and reviewers. We strongly encourage code deposition in a community repository (e.g. GitHub). See the Nature Portfolio guidelines for submitting code & software for further information.

Data

Policy information about availability of data

All manuscripts must include a data availability statement. This statement should provide the following information, where applicable:

- Accession codes, unique identifiers, or web links for publicly available datasets
- A description of any restrictions on data availability
- For clinical datasets or third party data, please ensure that the statement adheres to our policy

The data generated in this study are provided in the Source Data file as mentioned in the "Data availability" section of the manuscript. The custum made MATLAB code used for the behavioral and fiber photometry analysis is available on XXXX

Research involving human participants, their data, or biological material

Policy information about studies with human participants or human data. See also policy information about sex, gender (identity/presentation), and sexual orientation and race, ethnicity and racism.

Reporting on sex and gender	n/a
Reporting on race, ethnicity, or other socially relevant groupings	n/a
Population characteristics	n/a
Recruitment	n/a
Ethics oversight	n/a

Note that full information on the approval of the study protocol must also be provided in the manuscript.

Field-specific reporting

Please select the one below that is the best fit for your research. If you are not sure, read the appropriate sections before making your selection.

☒ Life sciences ☐ Behavioural & social sciences ☐ Ecological, evolutionary & environmental sciences

For a reference copy of the document with all sections, see nature.com/documents/nr-reporting-summary-flat.pdf

Life sciences study design

All studies must disclose on these points even when the disclosure is negative.

Sample size	We did not use statistical methods to pre-determine sample sizes.<br>Our sample sizes are similar to those reported in previous publications and the appropriate statistical comparisons were performed (Felix-Ortiz AC, Beyeler A, et al., Neuron (2013), Gehrlach DA, et al., Nat Neurosci (2019), Wang L, et al., Nature (2018)).
Data exclusions	Mice were excluded from the analysis when optic fiber, cannulae placement or viral expression patterns were out of brain targets.
Replication	Fiber photometry experiments: 3 experimenters collected the results for the aIC and pIC excitatory neurons recordings from 5 experimental replicates. For the aIC-BLA recordings, one experimenter collected the data from 3 experimental replicates.<br>Fiber photometry analysis: all experiments analysis was repeated by 3 independent researchers, and produced the same result.<br>Pharmacological experiments and analysis: 2 researchers made the analysis independently and found the same results.<br>Anatomical mapping: 2 experimenters collected the images, and 2 experimenters independently did the analysis and found the same result.<br>Optogenetics experiments: for the BiPOLES manipulations, 2 independent experiments were performed and combined. For the single inhibition or activation with GtACR and ChR2, each one was performed once.<br>Optogenetics analysis: one person performed the analysis of all experiments.<br>Histological verification: animal inclusion based on histological imaged were performed by 2 persons independently, for all experiments.<br>Electrophysiology ex vivo: data were collected by two different experimenters in 2 experimental replicates. All the analysis was performed by the second experimenter.
Randomization	We randomly assigned mice into study groups.
Blinding	For all behavioral analysis (fiber photometry recordings, pharmacological experiments and optogenetics) the experimenters were blind at the time of the recordings and analysis. For the ex vivo electrophysiological recordings, the experimenter was not blind to the neural type.

Reporting for specific materials, systems and methods

We require information from authors about some types of materials, experimental systems and methods used in many studies. Here, indicate whether each material, system or method listed is relevant to your study. If you are not sure if a list item applies to your research, read the appropriate section before selecting a response.

Materials & experimental systems

n/a	Involved in the study
☐	☒ Antibodies
☒	☐ Eukaryotic cell lines
☒	☐ Palaeontology and archaeology
☐	☒ Animals and other organisms
☒	☐ Clinical data
☒	☐ Dual use research of concern
☒	☐ Plants

Methods

n/a	Involved in the study
☒	☐ ChIP-seq
☒	☐ Flow cytometry
☒	☐ MRI-based neuroimaging

Antibodies

Antibodies used	anti-cFos (Synaptic System, #226-003), Alexa Fluor 555 Goat anti-rabbit IgG (Fischer scientific, #1890860),
Validation	Data sheet and website of Synaptic systems describes that anti-c-fos antibody was validated to react specifically with mouse c-fos, and has been widely used by over 40 papers. Moreover, this antibody was also used in a recent publication published in Nature Neuroscience. (Yamamuro K, et al., A prefrontal-paraventricular thalamus circuit requires juvenile social experience to regulate adult sociability in mice., Nature neuroscience (2020))

Animals and other research organisms

Policy information about studies involving animals; ARRIVE guidelines recommended for reporting animal research, and Sex and Gender in Research

Laboratory animals	Adult male and female C57BL/6J mice (Charles River Company) were aged 9-10 weeks at the beginning of the experiments.
Wild animals	This study did not use wild animals.
Reporting on sex	All experiments were performed in both males and female mice, and the number of male and female is systematically reported.
Field-collected samples	This study did not include field-collected samples.
Ethics oversight	All animal experiments in this study were conducted according to French governmental regulations, and approved by the ethical committee and the Ministry of Education, Research and Innovation (Saisine #16579) in accordance with the guidelines of the European Communities Council Directives.

Note that full information on the approval of the study protocol must also be provided in the manuscript.
